# Simvastatin Ameliorates Matrix Stiffness-Mediated Endothelial Monolayer Disruption

**DOI:** 10.1371/journal.pone.0147033

**Published:** 2016-01-13

**Authors:** Marsha C. Lampi, Courtney J. Faber, John Huynh, Francois Bordeleau, Matthew R. Zanotelli, Cynthia A. Reinhart-King

**Affiliations:** Meinig School of Biomedical Engineering, Cornell University, Ithaca, NY, United States of America; University of Illinois at Chicago, UNITED STATES

## Abstract

Arterial stiffening accompanies both aging and atherosclerosis, and age-related stiffening of the arterial intima increases RhoA activity and cell contractility contributing to increased endothelium permeability. Notably, statins are 3-hydroxy-3-methylglutaryl coenzyme A (HMG-CoA) reductase inhibitors whose pleiotropic effects include disrupting small GTPase activity; therefore, we hypothesized the statin simvastatin could be used to attenuate RhoA activity and inhibit the deleterious effects of increased age-related matrix stiffness on endothelial barrier function. Using polyacrylamide gels with stiffnesses of 2.5, 5, and 10 kPa to mimic the physiological stiffness of young and aged arteries, endothelial cells were grown to confluence and treated with simvastatin. Our data indicate that RhoA and phosphorylated myosin light chain activity increase with matrix stiffness but are attenuated when treated with the statin. Increases in cell contractility, cell-cell junction size, and indirect measurements of intercellular tension that increase with matrix stiffness, and are correlated with matrix stiffness-dependent increases in monolayer permeability, also decrease with statin treatment. Furthermore, we report that simvastatin increases activated Rac1 levels that contribute to endothelial barrier enhancing cytoskeletal reorganization. Simvastatin, which is prescribed clinically due to its ability to lower cholesterol, alters the endothelial cell response to increased matrix stiffness to restore endothelial monolayer barrier function, and therefore, presents a possible therapeutic intervention to prevent atherogenesis initiated by age-related arterial stiffening.

## Introduction

Age is a primary risk factor for atherosclerosis, and vascular stiffness increases with age due to changes in the extracellular matrix which include increased elastin fragmentation, collagen deposition, and collagen cross-linking by advanced glycation end products (AGEs) [1–4]. While the connection between macro-scale arterial stiffness and cardiovascular diseases is well characterized, the relationship between increased vessel stiffness and endothelium behavior on a cellular level is less clear [5,6].

Within the vasculature, endothelial cells maintain vascular homeostasis, in part, by forming a monolayer barrier along the arterial lumen. Endothelium integrity is dependent upon extracellular VE-cadherin interactions between adjacent cells and intracellular VE-cadherin anchoring to the actin cytoskeleton through catenins [7]. Cellular mechanotranduction occurs at both cell-matrix and cell-cell contacts [8]. Our group and others have shown that the mechanical stiffness of the cellular microenvironment plays a key role in dictating endothelial cell behaviors including cell area, adhesion, spreading, network formation, and sprouting [9–13]. Permeability of the endothelium is a key feature of atherosclerosis, as cholesterol flux across the vessel wall is an initiating step in atherogenesis [14–16]. Using *in vitro* and *ex vivo* models of vessel stiffness and aging, we previously showed that increasing substrate stiffness alone promoted RhoA/Rho-associated kinase mediated endothelial monolayer disruption and increased endothelium permeability [3]. RhoA-mediated actomyosin contractility is increased on stiff matrices, with increasing substrate stiffness leading to increased traction stresses [3,13,17,18]. Increased cellular traction stresses leads to the disruption of cell-cell junctions. As such, inhibition of cellular contractility is one potential avenue for the prevention of increased endothelial permeability in response to the matrix stiffening that occurs with age and atherosclerosis progression.

Interestingly, statins are 3-hydroxy-3-methylglutaryl coenzyme A (HMG-CoA) reductase inhibitors that are traditionally prescribed to lower blood cholesterol levels by inhibiting the production of the intermediate mevalonate during cholesterol synthesis, but are now recognized to have pleiotropic cardiovascular benefits [19–21]. Clinically, improvements in patient cardiovascular health that are not correlated to decreased cholesterol levels have been observed in as little as 4 weeks after initiating a statin regimen [22]. Statins improve vascular inflammation and reduce the risk of myocardial infarction and stroke [23,24]. Statins also reduce all-cause mortality in patients with and without histories of coronary artery disease [25,26]. It is now evident that inhibiting cholesterol biosynthesis with statins leads to aberrant activity of small GTPase signaling molecules. Mechanistically, it is well established that statins prevent the synthesis of isoprenoids that are post-translationally added to G-proteins [19–21] and it has been demonstrated that the addition of mevalonate or the isoprenoids directly rescues the effect of statins [27,28]. Within the Rho family of G-proteins, RhoA, Rac1, and Cdc42 are post-translationally prenylated with a geranylgeranyl pyrophosphate lipid anchor that is important for membrane localization, anchoring, and activation [29,30]. The statin, simvastatin, originally marketed by Merck under the brand name Zocor^®^, has been shown to attenuate RhoA activity and increase cytosolic activation of Rac1 by disrupting geranylgeranyl pyrophosphate synthesis to improve endothelial barrier function [28,31]. Notably, although also geranylgeranylated, altered Cdc42 activity does not contribute to the significant improvements in endothelial barrier function after simvastatin treatment [28].

In this study, we investigate the use of simvastatin to restore endothelial barrier integrity by altering pathways that contribute to increased RhoA-mediated cell contractility on stiff matrices. We also investigate Rac1 activity and cytoskeletal reorganization in response to simvastatin treatment. To date, previous studies have demonstrated that simvastatin pre-treatment attenuates barrier disruption caused by the known endothelial agonists thrombin and lipopolysaccharide [28,31,32], but have not accounted for physiological biomechanical stimuli such as extracellular matrix stiffness, which is altered with age and also disrupts the arterial endothelial barrier [3]. To investigate the effects of statin treatment on the disruption of endothelial barrier function due to matrix stiffness, we grew endothelial cell monolayers on polyacrylamide gels ranging in stiffness from 2.5 to 10 kPa to mimic the young and aged arterial intima respectively [33]. Our data indicate that simvastatin treatment alters the cellular response to substrate mechanics and attenuates increased RhoA activity and cell contractility caused by increased matrix stiffness to restore endothelial barrier integrity. Simvastatin also increased Rac1 activity and correlated with barrier enhancing cytoskeletal reorganization. These results indicate that using simvastatin treatment to interrupt pathways that affect RhoA and Rac1 activity may be one method to mitigate endothelium hyperpermeability that occurs in response to age-related arterial stiffening and prevent atherosclerosis.

## Materials and Methods

### Cell Culture and Polyacrylamide Gel Synthesis

Bovine aortic endothelial cells purchased from VEC Technologies (Rensselaer, NY) were used from passages 7–12. Endothelial cells were maintained at 37°C and 5% CO_2_ in Medium 199 (Invitrogen, Carlsbad, CA) with 10% Fetal Clone III (HyClone, Logan, UT), 1% MEM amino acids (Invitrogen), 1% MEM vitamins (Medtech, Manassas, VA), and 1% penicillin-streptomycin (Invitrogen) (complete Medium 199). Polyacrylamide (PA) gels with stiffnesses of 2.5, 5 and 10 kPa were made with bisacrylamide:acrylamide ratios of 5:0.1, 7.5:0.175, and 7.5:0.35, respectively. The PA gels and glass controls were coated with 0.1 mg/mL rat tail type I collagen (BD Biosciences), as described previously [3,9].

### Simvastatin Treatments

Simvastatin (Sigma-Aldrich) was activated as previously described [34–36]. Briefly, simvastatin prodrug was dissolved in 200-proof ethanol and incubated with 0.1 N NaOH for 2 hours at 50°C followed by the addition of MilliQ water. The solution was brought to a final pH of 7.0 using 0.1 N HCl and stored at 4°C. Simvastatin was diluted to final concentrations of 1 μM and 10 μM in complete M199 or L15 media for cell studies. Simvastatin treatment was 24 hours based on previous time course studies demonstrating greater effects on barrier enhancement with longer incubation times [28,37].

### RhoA and Rac1 Activity Assays

The colorimetric RhoA and Rac1 activity assays (Cytoskeleton #BK124 and #BK128) were carried out according to the manufacturer protocol. Lysate was collected from endothelial cell monolayers on 2.5, 5, and 10 kPa PA gels and glass controls treated with 0 or 10 μM simvastatin for 24 hours, two days post-confluence. The lysate from two gels at each condition was pooled and RhoA or Rac1 activity was normalized to the total protein content of the sample using the included Precision Red Protein Assay (#ADV02).

### Western Blotting

Two days post-confluence, endothelial cell monolayers on 2.5, 5, and 10 kPa PA gels and glass controls were treated with 0 or 10 μM simvastatin for 24 hours. Samples for phosphorylated myosin light chain (pMLC) analysis were lysed directly into boiling 2x Laemmli buffer, followed by immediate heating of the lysate at 95°C and heavy vortexing to disrupt nucleic acid structures. Lysate collected for the RhoA and Rac1 assays were used for quantifying total GTPase protein expression. Lysate was separated using sodium dodecyl sulfate polyacrylamide gel electrophoresis (SDS-PAGE) followed by protein transfer onto a polyvinyl difluoride (PVDF) membrane (Bio-Rad). Total RhoA was probed with a mouse monoclonal antibody against full-length RhoA (1:100) (Abcam, No. ab54835). Phosphorylated myosin light chain (pMLC) was probed with a polyclonal rabbit antibody against pMLC at threonine-18 and serine-19 (1:50) (Cell Signaling Technology, No. 3674). Total Rac1 was probed with a mouse monoclonal antibody against full-length Rac1 (1:00) (Millipore, No. 23A8). Alpha tubulin was probed as a loading control with a mouse polyclonal primary antibody (1:2000) (Sigma, No. T3559). Horseradish peroxidase (HRP) conjugated anti-rabbit and anti-mouse secondary antibodies were used (1:2000) (Rockland, No. 611-103-122 and Rockland, No. 610-103-121, respectively). The signal was developed with SuperSignal West Pico Chemiluminescent Substrate (Thermo Scientific). The membranes were exposed and imaged with a FujiFilm Image-Quant LAS-4000, followed by protein quantification using ImageJ software (v2.0.0-rc-41/1.50d, National Institutes of Health, Bethesda, MD, USA). The pMLC, RhoA, and Rac1 signals were normalized to the alpha tubulin loading control at each condition.

### Traction Force Microscopy

Endothelial cells were seeded on PA gels embedded with 0.5 μm diameter fluorescent beads (Invitrogen). Cells were allowed to adhere for 16 hours after which the media was removed and replaced with complete M199 containing 0 or 1 μM simvastatin. After 24 hours, phase contrast images of single cells were taken immediately followed by fluorescent images of the bead field beneath the cell. A second fluorescent image of the bead field in an unstressed state was taken after the cells were removed with trypsin/EDTA (Invitrogen). Images were acquired on a Zeiss Axio Observer.Z1m microscope equipped with a Hamamatsu ORCA-ER camera using a 20x objective. The magnitudes of traction force were calculated using the LIBTRC analysis library developed by M. Dembo (Dept. of Biomedical Engineering, Boston University) [38].

### Vinculin Focal Adhesion Quantification and Cell-Cell Junction Localization

Endothelial cells were seeded on 2.5, 5, and 10 kPa PA gels and allowed to adhere for 16 hours (single cells) or cultured 2 days post-confluence (monolayers). The growth media was then removed and replaced with complete M199 containing 0 or 1 μM simvastatin for 24 hours. Cells were fixed and permeabilized with 3.2% paraformaldehyde (EMS) and 1% Triton (VWR), respectively. Immunostaining was done with a mouse monoclonal vinculin antibody (1:100) (Santa Cruz, No. sc-59803) and a goat polyclonal VE-cadherin antibody (1:100) (Santa Cruz, No. sc-6458). Alexa Fluor 488 donkey anti-mouse (1:200) (Invitrogen, No. A21202) and Alexa Fluor 568 donkey anti-goat (1:200) (Invitrogen, No. A11057) secondary antibodies were used.

A z-stack image of each sample was captured using a Zeiss LSM700 microscope (v. 2010, Carl Zeiss MicroImaging GmbH, Jena, Germany) using a 40X/1.1 NA water immersion objective and 488 nm and 568 nm excitation laser lines, for vinculin and VE-cadherin, respectively. Images were then opened in ImageJ and converted into image sequences followed by automated image analysis performed with Matlab. To extract adhesion labeled structures, individual images were subjected to an adaptive Wiener filter (0.8 μm for vinculin and 0.6 μm for VE-cadherin filtering window) to remove background noise. Image sections presenting structures with a signal to noise ratio greater than 3:1 for vinculin and 2:1 for VE-cadherin where then subjected to a top-hat filter (1 μm diameter disk). Filtered images were further subjected to a median filter (0.48 μm filtering window) to correct for intensity variations while keeping necessary structures. To quantify FA length and area, confocal sections from vinculin stained single cells were used. To quantify vinculin:VE-cadherin overlap, corresponding vinculin and VE-cadherin stained endothelial cell monolayer confocal image stacks were filtered as described and then overlaid to generate the 3D overlapping volume data.

### Cell Circularity and Actin and Cortactin Arrangement

Two days post-confluence, endothelial cell monolayers on 10 kPa PA gels were treated with 0, 1, and 10 μM simvastatin for 24 hours. Cells were fixed and permeabilized with 3.7% formaldehyde (VWR) and 1% Triton (VWR), respectively. Immunostaining was done with a rabbit polyclonal cortactin primary antibody (1:100) (Santa Cruz, No. sc-11408) and Alexa Fluor 488 donkey anti-rabbit secondary (1:200) (Invitrogen, No. A21206). Actin was visualized with 594 FITC-conjugated phalloidin (1:100) (Invitrogen, No. A12381). Fluorescent images were captured on a Zeiss Axio Observer.Z1m microscope equipped with a Hamamatsu ORCA-ER camera using a 20x objective. Cell perimeters were outlined and cell circularity was calculated in ImageJ software where $circularity=\frac{4\pi \left(Area\right)}{{\left(Perimeter\right)}^{2}}$, and a perfect circle has a value of 1. Cortactin organization was quantified using MatLab code to quantify the number of linear segments at cell-cell junctions.

### Endothelial Permeability

Endothelial permeability was measured based on the flux of FITC-dextran through confluent endothelial cell monolayers into the underlying polyacrylamide gel, as described previously [3]. Two days post-confluence, endothelial cells seeded on PA gels cells were incubated in Leibovitz’s L15 media containing 10% FetalClone III (HyClone), 1% penicillin-streptomycin (Invitrogen) and either 0 or 1 μM simvastatin. After 24 hours, monolayer permeability was evaluated by adding a 10 μM, 40 kDa FITC-dextran (Sigma) solution to the cells for 10 minutes. For thrombin studies, 4 U/mL bovine thrombin (Calbiochem) was added for 5 minutes immediately prior to taking measurements. Confocal z-slice images were then acquired on a Leica TCS SP2 system equipped with a 40x dipping lens.

Endothelial permeability was quantified by measuring the fluorescent intensity above and below the cell layer. Briefly, the pixel intensity inside a 50 by 400 pixel box above the cell layer was recorded. Next, the pixel intensity within the gel was determined and recorded by drawing a 400 pixel wide box encompassing the entire height of the gel. This value represents the amount of FITC-dextran that permeated the cell monolayer into the gel over 10 minutes. Average permeability was calculated by dividing the average pixel intensity within the gel by the average pixel intensity above the gel. Gel permeability is dependent on stiffness; therefore to account for this effect, the average permeabilities were normalized against the permeability of control gels without cells at the same stiffness.

### Quantification of VE-cadherin Junction Gap Width

Two days post-confluence, endothelial monolayers on PA gels were treated with 0 and 1 μM simvastatin for 24 hours. Cells were fixed and permeabilized with 3.7% formaldehyde (VWR) and 1% Triton (VWR), respectively. VE-cadherin was visualized using a goat polyclonal VE-cadherin primary antibody (1:100) (Santa Cruz, No. sc-6458) and Alexa Fluor 568 donkey anti-goat secondary antibody (1:200) (Invitrogen, No. A11057). Fluorescent images were acquired on a Zeiss Axio Observer.Z1m microscope equipped with a Hamamatsu ORCA-ER camera using a 20x objective.

VE-cadherin junction width was quantified using ImageJ and a custom-written Matlab algorithm described previously [3]. Briefly, a line was drawn perpendicular to the cell junction in ImageJ to obtain a pixel intensity profile across each junction. The intensity profile was fit with a two-Gaussian curve in MATLAB and junction widths were defined as the width of the curve 20% above the baseline pixel intensity.

### Statistical Analysis

Statistical analysis was conducted in JMP v. 10. All data is mean ± SEM (standard error of the mean). Analysis of variance (ANOVA) followed by a Tukey’s Honestly Significant Difference (HSD) test were used for total RhoA expression, total Rac1 expression, traction force microscopy, focal adhesion size and length, cell circularity, cortactin arrangement, permeability, junction width, and vinculin localization analyses. Within stiffness changes in RhoA and Rac1 expression and vinculin localization were analyzed with a two-tailed Student’s t-test. Focal adhesion and vinculin localization data was log transformed to meet normality assumptions. Least squares means (LS-Means) regression was used to analyze Western blotting and RhoA-GTP activity using experiment as a random effect to account for differences in baseline values between experimental replicates. Rac1-GTP activity was analyzed using a linear mixed effects model to account for within experiment dependence.

## Results

### Simvastatin Alters the RhoA Pathway

To investigate the effect of simvastatin on endothelial barrier disruption associated with elevated RhoA activity caused by increased matrix stiffness, we probed RhoA activity and markers of endothelium integrity in response to both substrate stiffness and simvastatin treatments. We also investigated the effect of simvastatin on Rac1 activity, noting that Rac1 is reported to have inverse activation when compared to RhoA, and that Rac1 is also altered by simvastatin to improve endothelial barrier function [28,31,39].

**Fig 1:**
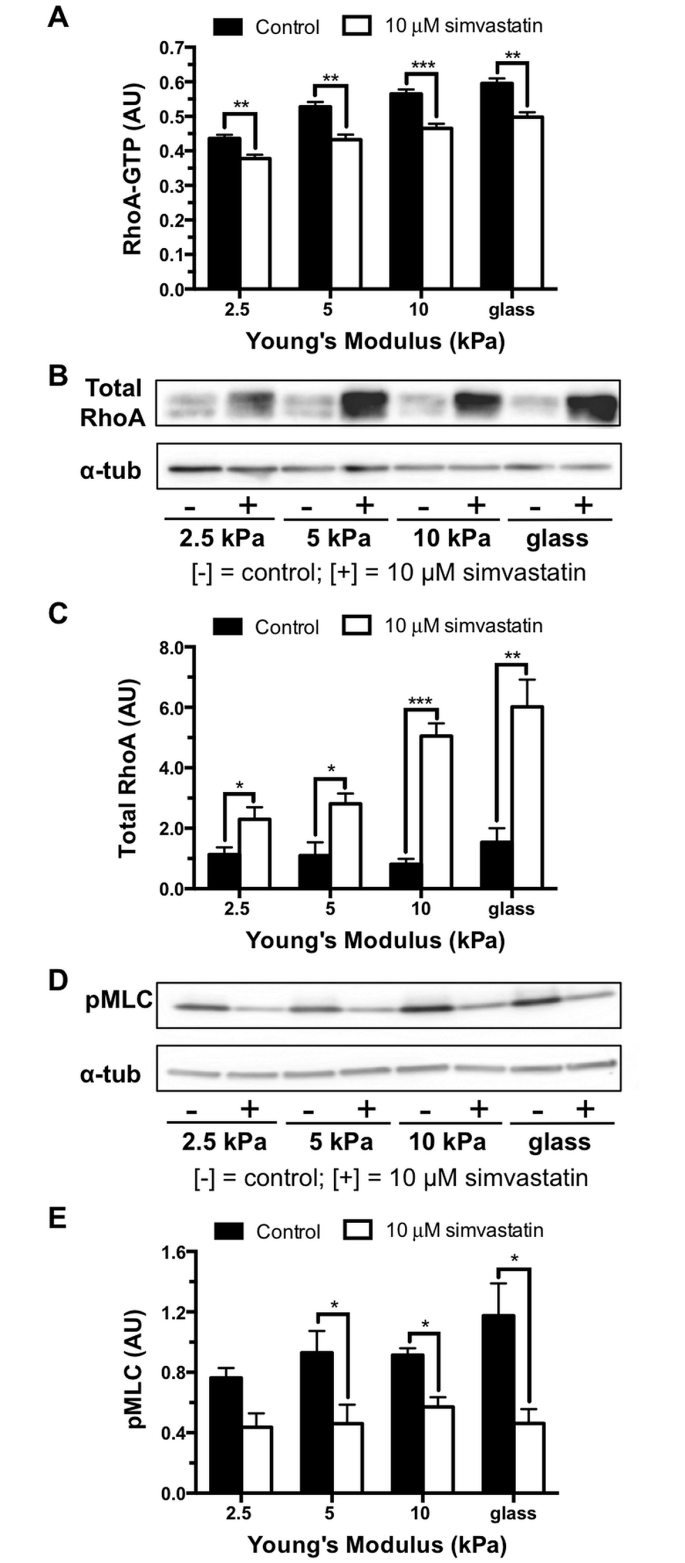
Simvastatin disrupts the RhoA pathway. The Rho/Rho-associated kinase pathway activity is elevated in endothelial monolayers with increased matrix stiffness but is attenuated with 10 μM simvastatin treatment. (A) Bar graphs of RhoA-GTP activity in response to matrix stiffness and simvastatin treatment normalized to total protein of lysate (n = 2, performed in triplicate). (B) Representative Western blot probing for total cellular RhoA expression and alpha tubulin (α-tub) loading control. (C) Quantification of total RhoA normalized to alpha tubulin loading control demonstrating that RhoA expression is significantly increased by the simvastatin treatment (n = 5). (D) Representative Western blot probing for phosphorylated myosin light chain (pMLC) and alpha tubulin loading control. (E) Western blot quantification normalized to alpha tubulin loading control demonstrating simvastatin attenuates increased pMLC caused by increased substrate stiffness, (n = 4). Data are presented as means ± standard error of the mean, *p<0.05, **p<0.01, ***p<0.001 when compared to the untreated control at each stiffness.

To modulate substrate stiffness and maintain physiological relevance, we grew bovine aortic endothelial cells on polyacrylamide gels with a lower stiffness level of 2.5 kPa to match the reported Young’s modulus of 2.7 ±1.1 kPa for the stiffness of the subendothelial matrix of healthy bovine carotid arteries [33]. Higher stiffness 5 and 10 kPa gels were used as models of aged arteries. As matrix stiffness increased, RhoA activity increased (Fig 1A). At each stiffness level, simvastatin treatment resulted in a significant decrease in active RhoA levels. Interestingly, while total cellular RhoA showed no significant change in expression over the stiffness range tested in control conditions, there was a robust increase in total RhoA across all stiffnesses with simvastatin treatment (Fig 1B and 1C). These results suggest a compensatory cellular response to the production of unprenylated RhoA and indicate the decrease in active RhoA-GTP caused by the statin treatment is not a result of lower expression levels. To further assess the effect of simvastatin on the RhoA/Rho-associated kinase contractility pathway, we probed endothelial monolayers for phosphorylated myosin light chain which is activated downstream of RhoA (Fig 1D). Similar to RhoA activity, activation of myosin light chain increased with stiffness, and the effect of matrix mechanics was attenuated with simvastatin treatment (Fig 1E). The significant decreases in active RhoA and phosphorylated myosin light chain levels suggest that simvastatin interferes with cell contractility pathways.

### Cellular Traction Forces Decrease with Simvastatin Treatment

**Fig 2:**
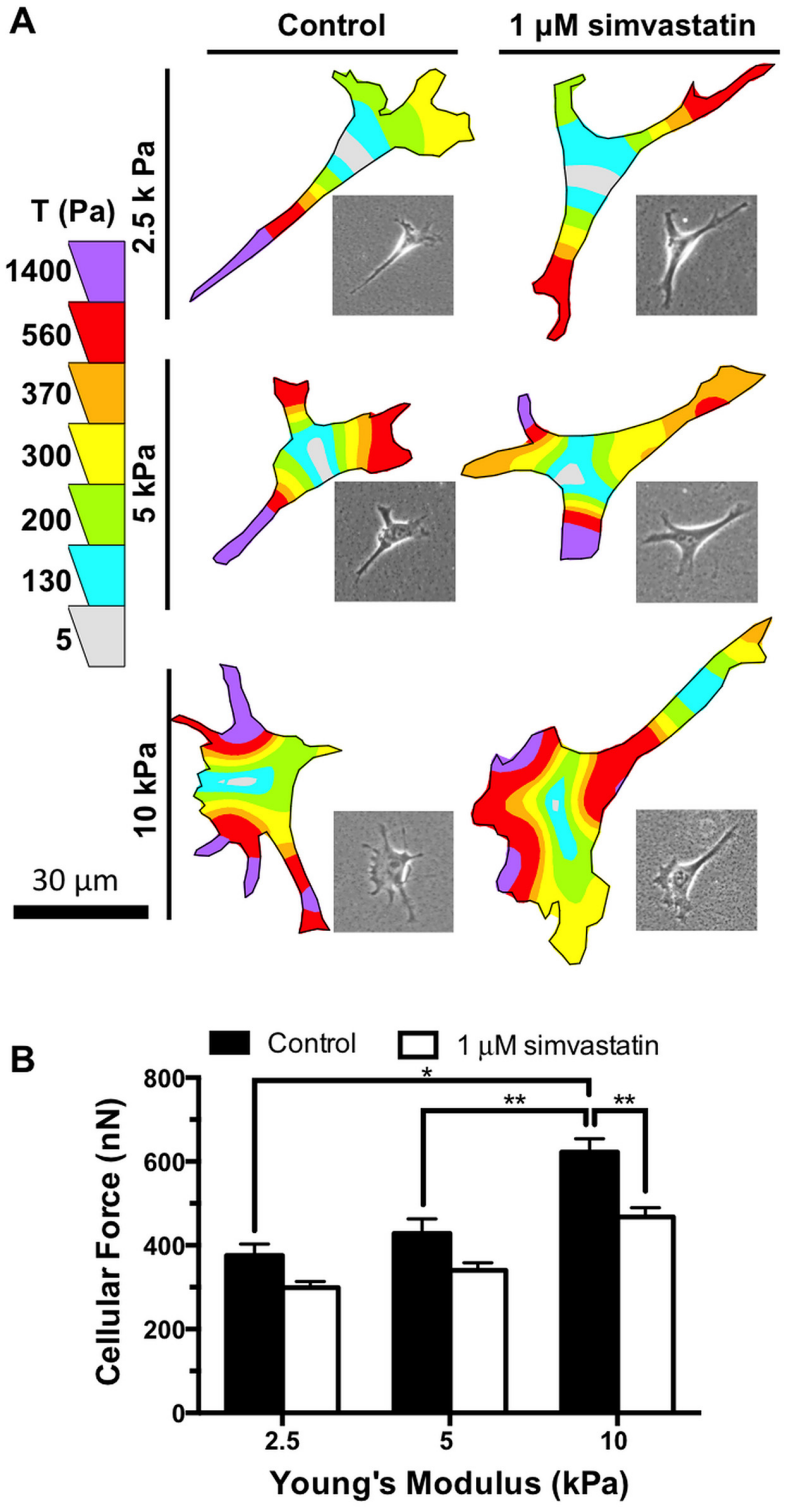
Simvastatin attenuates matrix stiffness-mediated traction forces. Cellular traction forces in endothelial cells increase with matrix stiffness but are attenuated with 1 μM simvastatin treatment. (A) Representative traction stress maps for endothelial cells at each level of matrix stiffness with and without simvastatin treatment. Inset shows corresponding phase image. (B) Cell contractility of endothelial cells increases with substrate stiffness but is attenuated by simvastatin (n = 3, 50–80 cells per condition). Data are presented as means ± standard error of the mean,*p<0.05, **p<0.01.

To directly assay for the effects of simvastatin on cell contractility, Traction Force Microscopy was used to quantify the contractile forces of endothelial cells seeded on matrices ranging in stiffness between 2.5 and 10 kPa that had been treated with simvastatin or control. We have previously shown that endothelial cell contractility increases with increasing substrate stiffness and can be attenuated by the addition of the Rho-associated kinase inhibitor, Y-27632 [3]. Therefore, we hypothesized that a decrease in active RhoA and pMLC caused by simvastatin interfering with RhoA post-translational modification would also significantly reduce cell contractility. Cellular force significantly increased with matrix stiffness for untreated endothelial cells (Fig 2A and 2B). After simvastatin treatment, cell contractility decreased at all stiffnesses tested when compared with the corresponding untreated condition. Most notably, there was a significant decrease in traction stresses after simvastatin treatment on the stiff, 10 kPa gels.

### Simvastatin Alters Endothelial Cell Focal Adhesions

**Fig 3:**
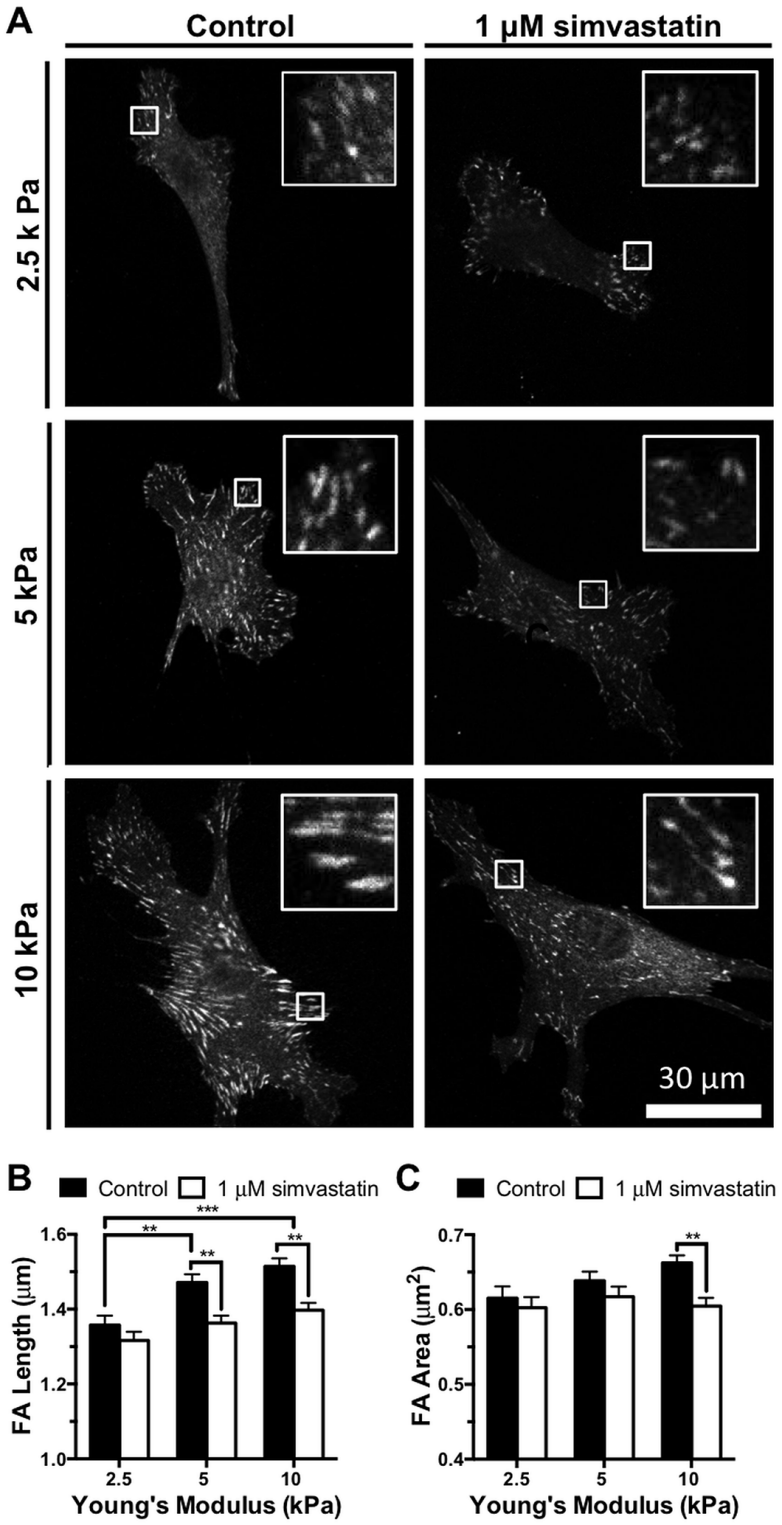
Simvastatin decreases endothelial cell focal adhesion length and area. Endothelial cell focal adhesions increase in length and area with matrix stiffness but decrease with 1 μM simvastatin treatment demonstrating that statins alter cell-matrix interactions. (A) Representative images of vinculin stained focal adhesions in individual endothelial cells with increasing matrix stiffness and 1 μM simvastatin treatment. Inset shows individual vinculin stained focal adhesions. (B) Focal adhesion (FA) length and (C) area increase with substrate stiffness but decrease with the statin treatment (n = 3, 38–56 cells per condition). Data are presented as means ± standard error of the mean,**p<0.01, ***p<0.001.

RhoA-mediated cell contractility is necessary for focal adhesion assembly [40] and the magnitude of traction force generated from an individual adhesion directly correlates to its size [41]; therefore, we treated endothelial cells with and without 1 μM simvastatin and stained for vinculin to quantify individual cell-matrix interactions using length and area as metrics (Fig 3A). As predicted based on our traction force data, focal adhesion length and area increased with increasing substrate stiffness, but were attenuated with simvastatin, most notably on the stiffer matrices (Figs 3B and 2C). Since focal adhesion formation relies on feedback loops transmitting matrix cues from integrins to the cytoskeleton [42], and it is established that stable focal adhesions are elevated on stiff matrices [43], our data demonstrate that simvastatin treatment is altering how mechanical signals from the underlying matrix are integrated into cellular responses.

### Simvastatin Alters Endothelial Cell Cytoskeletal Organization and Rac1 Activity

**Fig 4:**
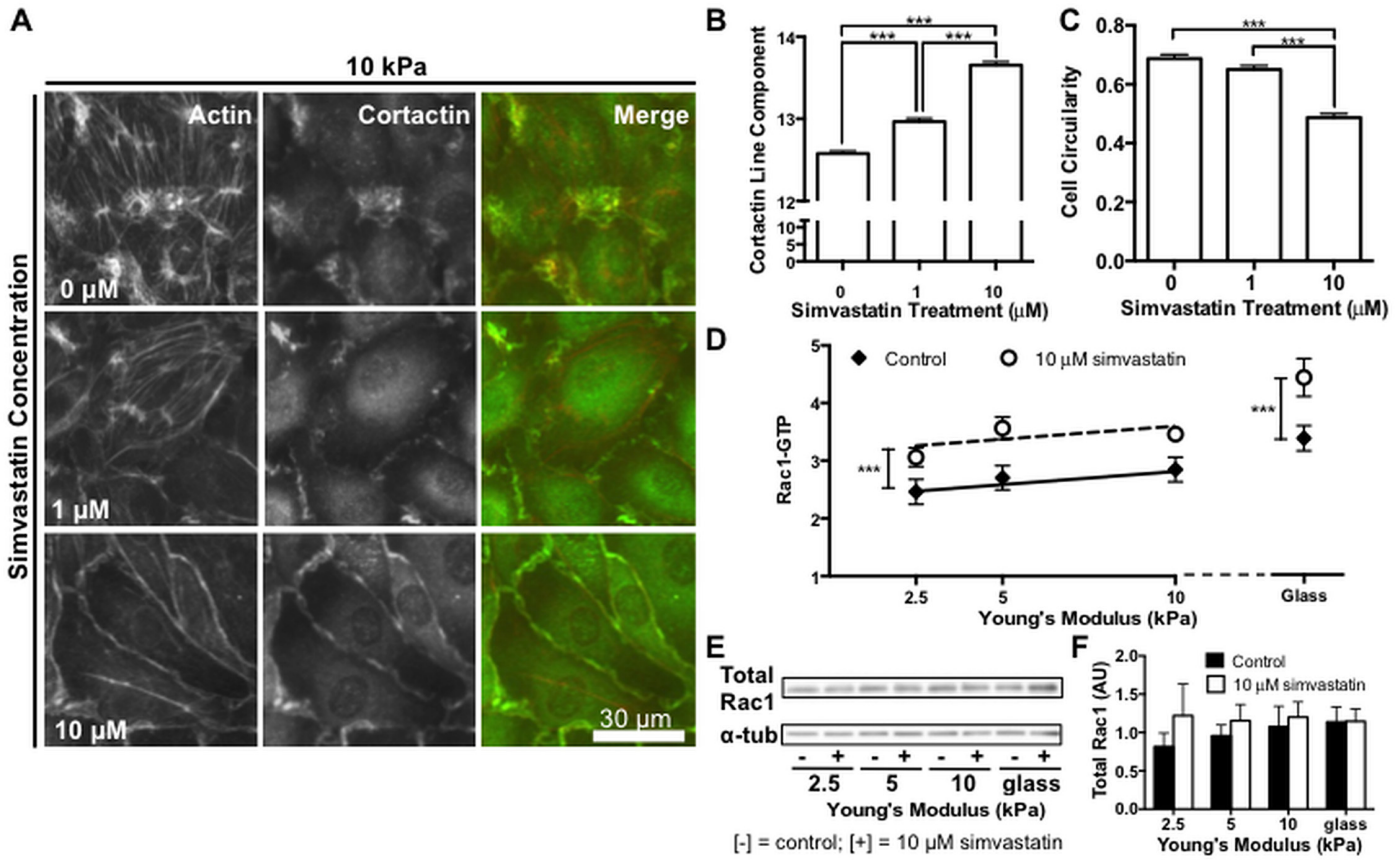
Simvastatin alters actin organization, cell morphology, and Rac1 activity in endothelial monolayers. Cytoskeletal organization and Rac1 activity in endothelial monolayers is altered by simvastatin treatment. (A) Representative images of endothelial monolayers showing prominent actin stress fibers in control cells and a barrier enhancing cortical actin ring that forms with increasing simvastatin concentration. Cortactin changes from puncta to organized linear segments around the cell periphery and localizes with actin with increasing statin treatment. (B) Cortactin organization, measured by quantifying linear segments at cell-cell junctions, increases with simvastatin (n = 3, 30 fields of view per condition). (C) Endothelial cells adopt an elongated morphology as actin stress fibers diminish with increasing simvastatin treatment. Cell circularity, where a perfectly circular cell has a value of 1, decreases with increasing simvastatin concentration (n = 3, 50–54 cells per condition). (D) Rac1-GTP activity normalized to total protein of lysate increases across all stiffness levels with simvastatin treatment (n = 5, performed in duplicate or triplicate). (E) Representative Western blot probing for total cellular Rac1 expression and alpha tubulin (α-tub) loading control. (F) Quantification of total Rac1 normalized to alpha tubulin loading control demonstrating no significant change in expression with stiffness or statin treatment (n = 5). Data are presented as means ± standard error of the mean, ***p<0.001.

Since simvastatin had the greatest effect on cell contractility and focal adhesion formation on stiff matrices, and transverse actin stress fibers are used to exert contractile forces [44] we investigated the effects of simvastatin on the cytoskeleton using 10 kPa polyacrylamide gels where the highest contractile forces were observed. Previous work has shown that enhanced endothelial integrity by simvastatin treatment has been associated with distinct patterns of actin reorganization from stress fibers into an endothelial barrier enhancing cortical actin ring [31]. The formation of a cortical actin ring has also been associated with known barrier enhancing stimuli such as shear stress and sphingosine-1-phospate (S1P) [45,46]. As such, we were interested in whether simvastatin could attenuate the formation of prominent actin stress fibers and instead promote a barrier protective phenotype on stiff matrices. Endothelial cell monolayers on 10 kPa polyacrylamide gels were treated with 0, 1, or 10 μM simvastatin and fluorescently stained for actin (Fig 4A). Consistent with our contractility data, we observed prominent actin stress fibers in the control monolayers that decreased in a dose-dependent manner with simvastatin treatment. At higher simvastatin concentrations, a cortical actin ring defined cell-cell junctions suggesting improved barrier integrity. Cortactin activates the Arp 2/3 complex for actin assembly [47] and its translocation to the cell periphery is necessary for cortical actin organization resulting in improved endothelial barrier integrity by S1P [46]. Similarly, cortactin translocation to cell edges has been reported in endothelial cells after simvastatin treatment [31]. Therefore, we fluorescently stained endothelial cell monolayers treated with 0, 1, or 10 μM simvastatin to determine if cortactin translocation could be an underlying mechanism mediating the barrier protective cytoskeletal reorganization we observed on stiff matrices in response to simvastatin (Fig 4A). Whereas cortactin in control cells was found in concentrated puncta around the cell periphery, it was linearly organized along the entire perimeter in cells treated with 10 μM simvastatin. Cortactin reorganization, measured by quantifying the number of linear segments along the cell perimeter, significantly increased with the statin treatment (Fig 4B). Cytoskeletal changes were accompanied by a distinct change in cell shape to a more elongated morphology with increasing statin treatment. The change in cell morphology was quantified using cell circularity where a perfectly circular cell has a value of 1 (Fig 4C).

Since Rac1 has been identified as the upstream effector mediating cortactin translocation [48], and it is also a prenylated G-protein that regulates cytoskeletal dynamics in concert with RhoA [39], we investigated whether simvastatin was also altering Rac1 activity. While there was no significant difference in activated Rac1 with increasing physiological matrix stiffness, across all stiffnesses, endothelial cell monolayers receiving the statin treatment exhibited increased active Rac1-GTP levels that were consistent with the cortactin translocation we observed (Fig 4D). Expression of total cellular Rac1 was unchanged by either matrix stiffness or simvastatin treatment (Fig 4E and 4F). Interestingly, although both RhoA and Rac1 are post-translationally modified with a geranygeranyl moiety, our results demonstrate that simvastatin differentially affects RhoA and Rac1 pathways to alter downstream indicators of cellular mechanosensing such as cytoskeletal organization, stress fiber formation, and contractility.

### Simvastatin Decreases Endothelium Permeability

**Fig 5:**
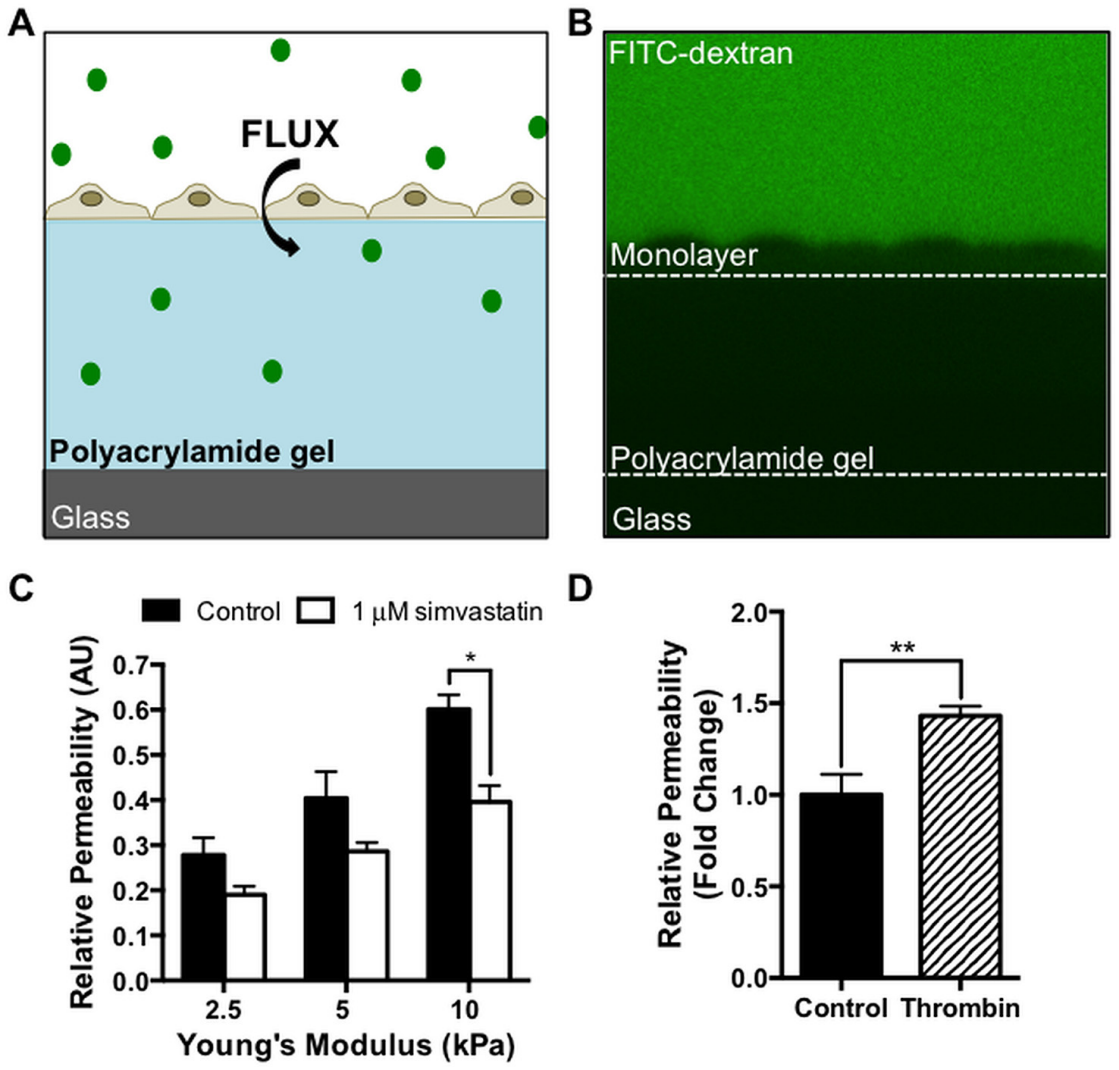
Simvastatin restores endothelial barrier function on stiff matrices. Endothelial monolayer permeability is attenuated with simvastatin treatment. (A) Schematic drawing of the *in vitro* permeability system where the flux of a 40-kDa FITC-dextran across an endothelial monolayer into the underlying polyacrylamide gel is measured. (B) Representative experimental X-Z image illustrating the fluorescent FITC-dextran solution, the endothelial monolayer, and the polyacrylamide gel. (C) Endothelium permeability increases with increasing substrate stiffness, but is significantly reduced with 1 μM simvastatin at the 10 kPa stiffness level (n = 2, 7–10 monolayers per condition). (D) Endothelial permeability on 10 kPa gels increases with the agonist thrombin (n = 4, 6–10 monolayers per condition). Data are presented as means ± standard error of the mean,*p<0.05, **p<0.01.

To directly assay if simvastatin attenuated the effects of increased matrix stiffness on endothelium barrier function, we measured permeability based on the flux of a 40 kDa fluorescein isothio-cyanate (FITC)–dextran across the endothelial monolayer into the polyacrylamide hydrogel below the cells (Fig 5A and 5B). The dextran size was chosen to match the hydrodynamic radius of albumin, a model protein used in vascular permeability studies [49,50]. Following simvastatin treatment, endothelial monolayers grown on 10 kPa gels exhibited significantly lower permeability compared to monolayers that did not receive the statin treatment (Fig 5C). At lower stiffnesses of 2.5 and 5 kPa, permeability also decreased with treatment, although the effect was less pronounced. Permeability on 10 kPa PA gels where we observed the greatest improvement in barrier function with simvastatin was validated using thrombin (4U/mL) as a positive control (Fig 5D).

### Simvastatin Decreases Cell-Cell Junction Size in Endothelial Monolayers

**Fig 6:**
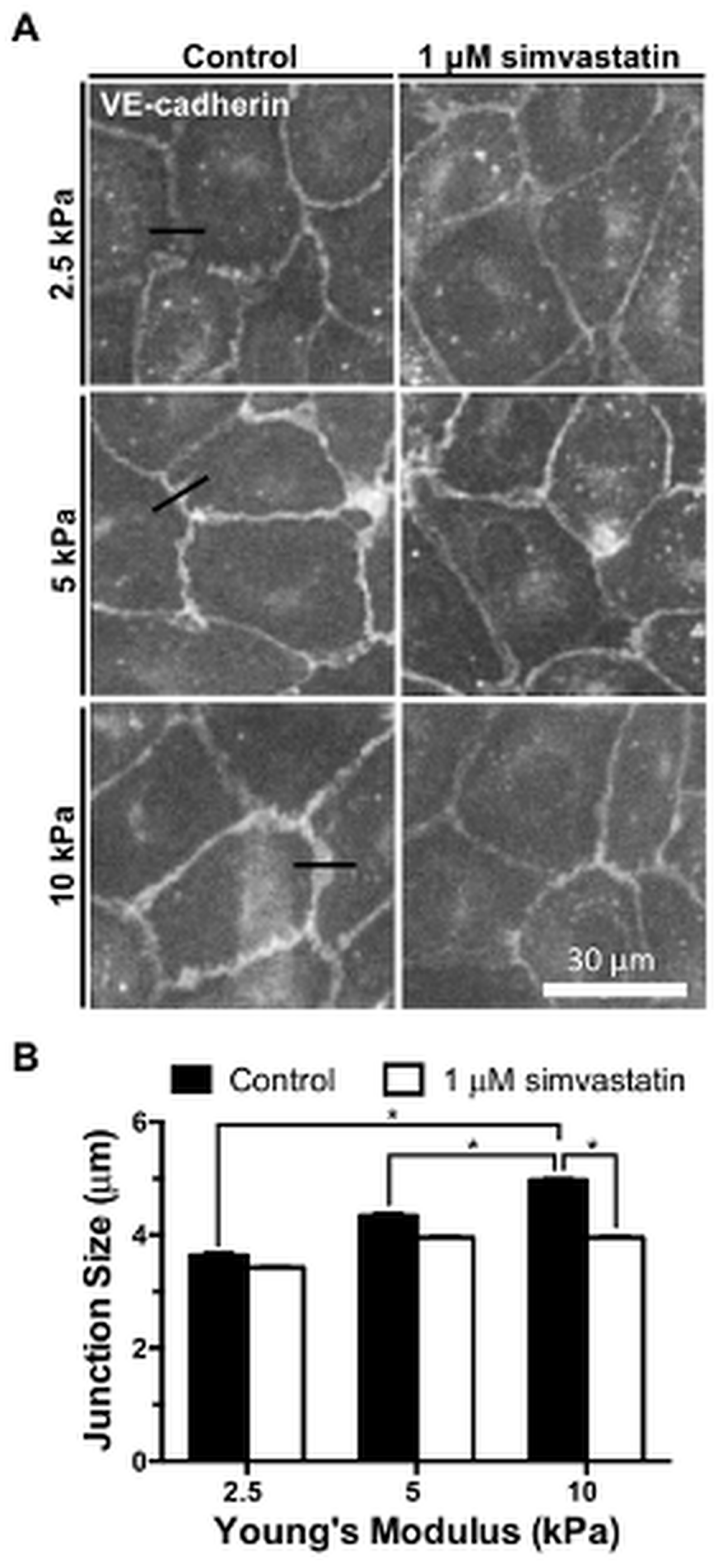
Increased endothelial cell-cell junction size on stiff matrices is diminished after simvastatin treatment. Endothelial monolayers are stained for VE-cadherin and cell-cell junction sizes are measured with control and 1 μM simvastatin treatments. (A) Representative images of fluorescently stained VE-cadherin monolayers indicating where junction width measurements were made (black line). (B) Cell-cell junction size increases with matrix stiffness but decreases with simvastatin (n = 2, 100–200 junctions per condition). Data are presented as means ± standard error of the mean,*p<0.05.

Noting that simvastatin altered endothelial permeability and that VE-cadherin is a critical factor for cadherin junction integrity that also interacts with the cytoskeleton, we investigated endothelial junction integrity as a function of stiffness in response to statin treatment. Endothelial monolayers seeded on polyacrylamide gels of varying stiffness were treated with simvastatin or control and stained for VE-cadherin (Fig 6A). Endothelial cell separation was measured by drawing a line perpendicular to the cell-cell junction at its widest point, and junction size was calculated by fitting the pixel intensity profile to a double Gaussian curve. Junction widths were measured 20% above the baseline pixel intensity [3]. Cell-cell junction size increased with substrate stiffness, and was significantly decreased with the simvastatin treatment on 10 kPa substrates (Fig 6B), restoring junction dimensions to those found on compliant (2.5 kPa) gels. These data suggest that simvastatin improves endothelial barrier functionality on stiff matrices by altering VE-cadherin cell-cell adhesions.

### Simvastatin Attenuates Intercellular Tension in Monolayers

**Fig 7:**
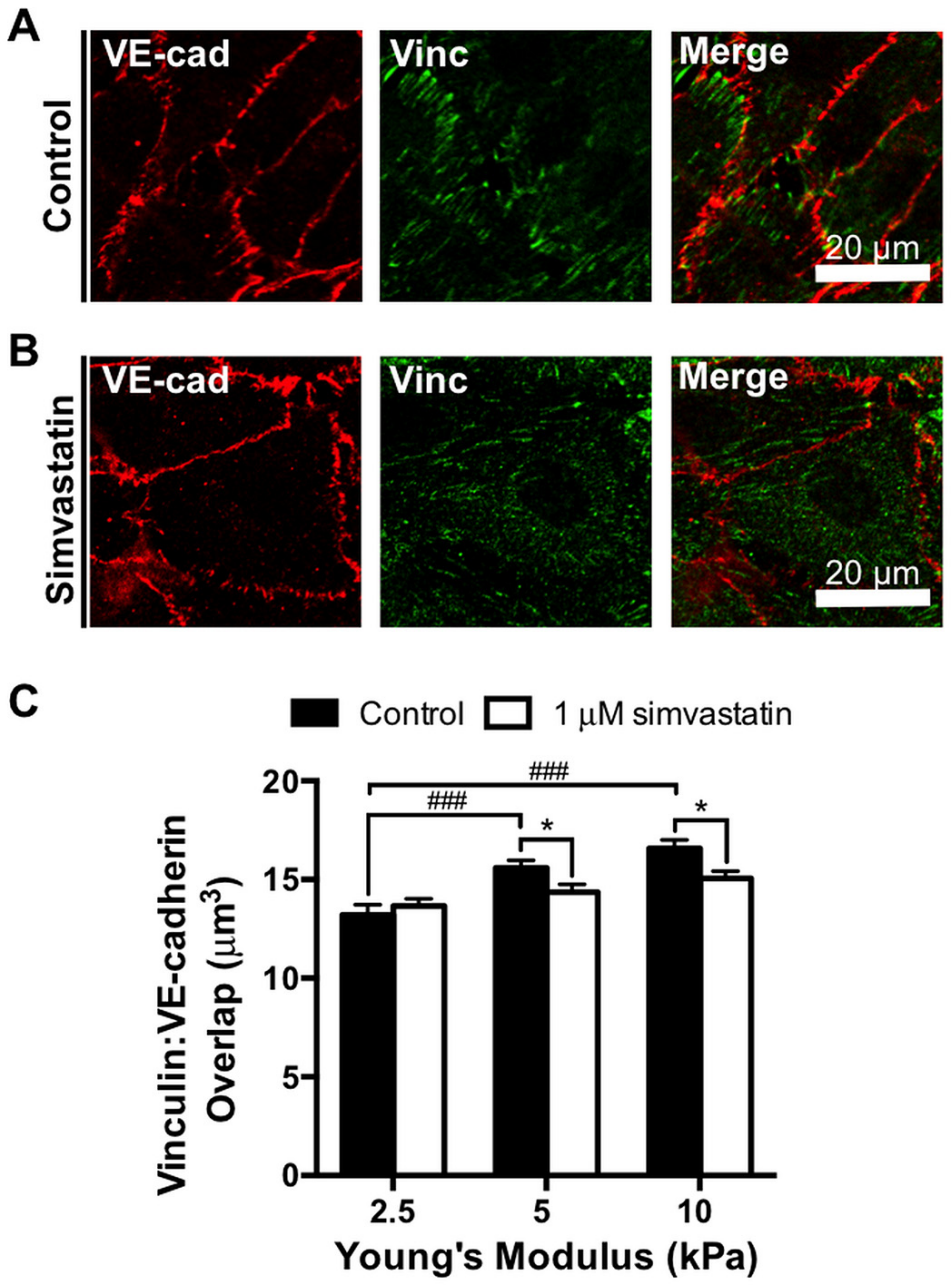
Simvastatin reduces vinculin localization at cell-cell adhesions in endothelial monolayers. Endothelial monolayers treated with control or 1 μM simvastatin are stained for vinculin and VE-cadherin, and the vinculin volume overlapping with VE-cadherin is quantified. Representative images of endothelial cell-cell junctions within a confluent monolayer fluorescently stained for vinculin and VE-cadherin on a 10 kPa polyacrylamide gel after 24 hour (A) control or (B) 1 μM simvastatin treatment demonstrating vinculin positive and vinculin negative junctions, respectively. (C) Vinculin localization per monolayer at cell-cell adhesions, a readout of intercellular junction tension, is quantified and increases with matrix stiffness but is significantly decreased with the statin treatment at higher matrix stiffnesses (n = 3, 70–90 fields of view per condition). Data are presented as means ± standard error of the mean, ###p<0.001 compared to matrix stiffness, *p<0.05 compared to the untreated control.

To further characterize the role of simvastatin in attenuating the effect of increased matrix stiffness on cell-cell junctions in endothelial monolayers, we used the mechano-sensitive protein vinculin as a readout of intercellular junction tension. It has been established that vinculin localizes at adherens junctions under tension and during remodeling, but is absent from mature cell-cell contacts [51,52]. Furthermore, Huveneers *et al*. demonstrate that stable endothelial junctions are converted into remodeling, vinculin positive junctions after adding thrombin, thereby connecting endothelium permeability to vinculin localization and cell-cell junction tension [52]. Endothelial monolayers grown on polyacrylamide gels of increasing stiffness were treated with control or 1 μM simvastatin and fluorescently stained for vinculin and VE-cadherin (Fig 7A and 7B). Vinculin localization was measured using confocal microscopy and automated image analysis to quantify the volume of vinculin per monolayer overlapping with VE-cadherin at cell-cell junctions. Increased matrix stiffness correlated with a significant increase in vinculin at cell-cell contacts that is decreased at higher stiffnesses with simvastatin treatment (Fig 7C). These data suggest that cell-cell adhesions within endothelial monolayers on stiff matrices are under increased tension and are less-stable, but have improved integrity after simvastatin treatment [52–54].

Collectively, our data suggests that simvastatin may provide age-related atheroprotective benefits by altering pathways that disrupt RhoA and Rac1 activity to contribute to restored endothelium integrity and barrier function on stiff matrices.

## Discussion

To date, there are no FDA-approved therapeutics to reverse age-related vessel stiffening even though arterial stiffness is well accepted to occur with age and is a negative predictor of cardiovascular health [55,56]. Stiffening of the arterial intima disrupts endothelium integrity, and the flux of cholesterol across the endothelium is the first-step in atherogenesis, suggesting that maintaining or restoring endothelial barrier function may be one method to mitigate atherosclerosis [3]. Notably, the small GTPase RhoA is known to play a critical role in disrupted endothelial adherens junctions and its activity is elevated in response to increased matrix stiffness [3,17,57]. Here, we demonstrate the use of simvastatin to inhibit the endothelial cell response to increased substrate stiffness and show that simvastatin alters RhoA and Rac1 pathways to restore endothelial monolayer integrity.

An altered endothelium is known to precede atherosclerotic lesion development [58], and increased collagen crosslinking by advanced glycation end products (AGEs) is a major contributor to stiffening of the vascular wall [59]. Therapeutics to reverse arterial stiffening by breaking AGE crosslinks or preventing AGE formation have been met with limited success despite reaching clinical trials. Fasudil, the FDA approved Rho-associated kinase inhibitor, has shown promise in treating atherosclerosis, however because of its high potency, it is unlikely to be widely administered to prevent cardiovascular disease [60,61]. Therefore, new approaches to target vascular stiffening or the cellular response to stiffening are required. Our data suggest that statins may be one approach to preventing the aberrant endothelial cell response to vascular stiffening.

Numerous pleiotropic effects of statins can be attributed to the inhibition of isoprenoid production which disrupts post-translational prenylation of the Rho family of GTPases: RhoA, Rac1, and Cdc42. Specific attention has been placed on RhoA because of its role in regulating contractility of the actin cytoskeleton and the importance of post-translational prenylation for its localization and activity at the cell membrane [57]. In agreement with other studies, we report that simvastatin decreases RhoA activity and results in improved endothelial barrier function. Furthermore, we report elevated Rac1 activity after the statin treatment that is correlated with barrier enhancing cytoskeletal rearrangements. This paradoxical activation of Rac1 has been previously attributed to the inability of unprenylated Rac1 to associate with its GDI [62], upstream activation of the AMP-activated protein kinase (AMPK) pathway [63], and the inverse activation relationship between RhoA and Rac1 [39]. To date, improved barrier integrity by simvastatin has not been associated with altered Cdc42 activity, and therefore, was not included in this study [28]. The pleiotropic effects of statins are not limited to altering the RhoA family of GTPases and there is significant crosstalk between cellular mechanotransduction pathways that continue to be elucidated, therefore, we acknowledge that simvastatin may be acting through additional pathways to ameliorate the effects of increased matrix stiffness (for reviews on the pleiotropic effects of statins see references [19–21]. While other groups have shown the efficacy of simvastatin in response to exogenous chemical agonists such as thrombin or in diabetic disease models, we are the first to show that statins can counteract the deleterious effects of matrix stiffening that occurs with normal aging [28,31]. Our results suggest that statins have the potential to benefit otherwise healthy adults as they age by maintaining endothelial barrier integrity to prevent the onset of atherosclerosis and subsequent cardiovascular diseases.

RhoA activity has generally been regarded as detrimental to endothelial barrier function [57]; however, basal RhoA activity is required for intact endothelial junctions and VE-cadherin expression [64]. Szulcek and colleagues reported its role in promoting the formation of endothelial cell-cell junctions [65]. Hotspots of RhoA activity were identified in regions of the cell membrane where endothelial protrusions were creating new junctions after spontaneous small gap formation. Interestingly, coordinated RhoA activity is also necessary for cell polarization and alignment in response to shear stress, hallmarks of a healthy endothelium [66,67]. Similarly, Rac1 can have barrier protective or disruptive effects depending on its specific localization and activation. Membrane bound Rac1 can produce deleterious reactive oxygen species at cell-cell adhesions [68], but also stabilizes adherens junctions by retaining VE-cadherin [69]. Cytosolic Rac1, in agreement with our data, is necessary for cortactin shuttling and cortical actin polymerization [27]. Since precise regulation of RhoA and Rac1 are necessary for endothelial functionality, developing barrier-restoring therapeutics presents a particular challenge and should not focus on complete suppression or amplification of their activity.

Endothelial cells are modulators of vascular homeostasis and exhibit improved functionality after statin treatment. Notably, endothelial cells are also mechanosensitive, incorporating matrix signals from basal integrins and flow stimuli from the apical glycocalyx [70,71]. The cross talk between mechanical cues and statin treatment is not well understood. Previous work by others has shown that a synergistic response between statins and mechanical force exists, where laminar shear stress with statin treatment provided enhanced protection against oxidative stress. However, under disturbed flow conditions, the protective benefit of the statin was diminished [72]. This prior work lays the foundation to suggest that there is an intersection in the mechanotransductive effects of shear stress and the signaling pathways affected by statin treatment. Our data furthered this work by demonstrating that other mechanical cues, specifically increased matrix stiffness, also exhibit crosstalk with the pathways targeted by statins.

Cardiovascular stiffening is well known to occur with age, and the role of vascular stiffening as a cause rather than a consequence of cardiovascular diseases has been demonstrated in several recent studies [55]. Weisbrod and colleagues recently demonstrated that micro-scale vessel stiffening preceded hypertension in a mouse model of diet-induced obesity [73], and clinical trials have shown that macro-scale arterial stiffening also precedes hypertension [74]. Our group has shown that intimal stiffness causes endothelial barrier disruption due to increased RhoA-mediated cell contractility, and that the endothelial response to increased matrix stiffness, and not adhesion molecule expression, contributed to enhanced leukocyte transmigration [3]. Moreover, endothelial monolayers cultured on stiff matrices, are unable to recover from leukocyte transmigration events leading to persistent gaps in the endothelial layer [75], through which subsequent leukocytes can transmigrate at increased rates [76]. *In* vivo, delayed repair of the endothelium could promote a local inflammatory response and drive atherosclerotic plaque formation. These recent advances in the literature indicate that vascular stiffening may be a potential target to mitigate cardiovascular disease pathologies associated with a compromised endothelium. As such, preventing the aberrant cellular response to age-related arterial stiffening to restore endothelial barrier function using statins, for example, as was done here, may be a promising approach to inhibiting atherosclerosis.
